# Construction, characterization, and bioavailability evaluation of honokiol-loaded porous starch by melting method without any solvent

**DOI:** 10.1080/10717544.2021.2009938

**Published:** 2021-12-06

**Authors:** Weiwei Wu, Haiyang Xu

**Affiliations:** School of Basic Medical Sciences, Shandong First Medical University & Shandong Academy of Medical Sciences, Taian, China

**Keywords:** Honokiol, porous starch, solubility, bioavailability

## Abstract

In the present study, the porous starch (PS) was used as an efficient carrier of honokiol (HK), and the HK-loaded PS (HPS) delivery system was prepared by melting method without using organic solvents. Its physical-chemical properties, solubility and oral bioavailability were also investigated. The obtained results proved that the HK in the HPS was mostly amorphous when it was loaded into the PSs with 87.54 ± 1.52% of encapsulation efficiency (EE) and 12.51 ± 0.22% of drug loading (DL) capacity. The water-solubility of the HPS was increased to 115.27 ± 2.92 μg/mL (pH = 1.2, artificial gastric juice (AGJ)), 161.58 ± 3.42 (pH = 6.8, artificial intestinal juice (AIJ)) and 148.5 ± 1.89 μg/mL (pH = 5.5, simulated tumor microenvironment), being 6.07, 4.38 and 4.87-folds higher than free HK. *In vitro* dissolution tests showed the HK was significantly higher from HPS than from free HK. Furthermore, compared with free HK, the release rate and the bioavailability was also substantially improved for HK from the HPS. Meanwhile, the HPS generated a higher inhibition to HepG2 cells than free HK.

## 1. Introduction

**Figure 1.**
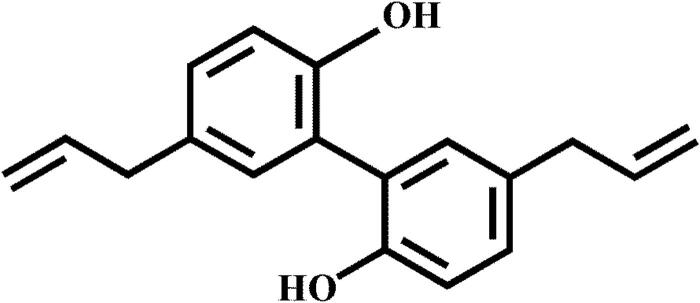
Chemical structure of honokiol.

Honokiol (HK, Figure 1), a bioactive lignanoid isolated from *Magnolia officinalis* or other species of Magnoliaceae that has been used for centuries in traditional Chinese and Japanese medicine in a variety of ways: to assuage anxiety, to treat thrombotic stroke, and to mitigate gastrointestinal discomfort (Zhang et al., 2015; Cheng et al., 2016). Furthermore, the HK exhibits several pharmacological properties, such as anti-cancer effects, anti-inflammatory effects, anti-oxidant actions, and anti-anxiety effects (Wang et al., 2011; Han et al., 2014; Xu et al., 2014; Zhang et al., 2015). However, this molecule’s poor solubility limits its dispersion in an aqueous solution, greatly restricting its applications.

Recently, starch has become a new biomaterial with potential pharmaceutical value. The biopolymer has distinctive physicochemical and functional properties and has the advantages of low price, nontoxicity, pure separation from plant sources, good biocompatibility, biodegradability, and interaction with living cells (Sujka et al., 2018). Porous starch (PS) is obtained by physical, chemical, and enzymatic modification of starch (Zhang et al., 2012; Majzoobi et al., 2015; Benavent-Gil & Rosell, 2017). It contains abundant pores or hollows that are formed from the surface to the center of the granules, resulting in a stable pore structure and high pore volume, and specific surface area. It is an excellent natural absorbent, which has been widely used in food, cosmetics, and other related industries so far (Zhu et al., 2019). In addition, the PS is also an ideal drug delivery system because of its advantages of biocompatibility, bioadhesive, and capable of high drug loading, and the PS is officially accepted by all major regulatory agencies for use in various oral drug delivery systems (Wu et al., 2011). So far, the PS has been applied to many lipophilic drugs, such as lovastatin (Wu et al., 2011), nitrendipine (Zhao et al., 2016), carbamazepine (Ali et al., 2013), and ketoprofen (García-González et al., 2012), etc., so as to improve its solubility and absorption *in vivo*.

The commonly used methods of preparing drug-loaded PS systems included spray drying and solvent removal methods (centrifugation or evaporation) and so on (Zhang et al., 2013; Pawar et al., 2017). These methods are simple and easy, but usually require organic solvents to dissolve the drugs in the preparation process, and the use of organic solvents can easily contaminate the environment and the residual solvents may cause some harm to human bodies. Therefore, in this paper, we attempted to prepare an HK-loaded PS system by the melting method without using organic solvents. The melting method is a clean and nonpolluting technology that does not use dangerous acid and base solutions or organic reagents. The preparation process is simple, the operation conditions are easy to control, and the product has good reproducibility. In addition, our group has tried to load melatonin into the PSs and use acetone as the solvent of the drug, and the EE of the prepared delivery system was about 70.17%. However, in this study, the EE of the delivery system prepared by the melting method was about 87.54%, which was higher than that prepared by the solvent removal method (Li et al., 2018). Furthermore, the preparation of a drug-loaded PS system by the melting method has not been reported until now.

This work was carried out to explore the PS as a carrier for insoluble HK, by using the advantages of the stable pore structure and high pore volume and specific surface area of PS in order to increase the solubility of HK and improve its bioavailability. In this experiment, the HPS systems were prepared by the melting method, and their physicochemical properties were characterized by scanning electron microscopy (SEM), X-ray diffraction (XRD), Fourier-transform infrared (FTIR) spectroscopy, and differential scanning calorimetry (DSC). Furthermore, the saturation solubility, the dissolution *in vitro*, the bioavailability *in vivo* and the antitumor activity were also investigated and analyzed.

## 2. Materials and methods

### 2.1. Materials

Honokiol (98.5% pure) was purchased from Baoji Haoxiang Biotechnology Co., Ltd. (Shanxi, China); Insoluble porous starch (corn starch treated with α-amylase and glucoamylase in weak acid)was obtained from Liaoning Lida Bio-technology Co., Ltd., (Liaoning, China); The glucoamylase (1.0 × 10^6^ μ/g) and α-amylase (4000 μ/g) were purchased from Yuanye Biotechnology Co., Ltd., (Shanghai, China).

### 2.2. Preparation of the HK-loaded PS system

The HK (1 g) and the PS (5 g) were accurately weighed and gently mixed for 5 min. The obtained mixture was placed in the oven at 90 °C (The melting point of HK was about 87.3 °C) so that HK was fully melted. After a period of time, the mixture was immediately transferred to the refrigerator at −40 °C for 2 h to cool and solidify. The HK-loaded PS system (HPS) was obtained and used for the following tests.

In addition, in the preparation processes, the mass ratio of PS to HK and the melting time can affect the solubility of the HPS samples. Therefore, the single-factor experiment was operated to investigate the effects of the two factors on the solubility of the HPS, so as to obtain the optimal operating conditions. The specific optimization process is described in the supplementary material section

### 2.3. Characterization of the HPS

#### 2.3.1. Scanning electron microscopy (SEM)

The granule morphology of free HK, the HPS, the PS, and the physical mixture (PM) of HK with PS was observed using an SEM (FEI, Eindhoven, Netherlands). Samples were coated with gold in a vacuum evaporator prior to observation. The obtained samples were examined at an accelerating voltage of 10 kV. The total ray counting rate range, dead time, and count time were 2000–3000 cps, <30%, and 100 s, respectively.

#### 2.3.2. Fourier-transform infrared spectroscopy (FTIR)

FTIR scan of free HK, the HPS, the PS, and PM of HK with PS was performed on an FTIR spectrophotometer (SHIMADZU, Kyoto, Japan). All samples were blended with solid KBr powder, and transmittances were recorded at wavenumbers between 4000 cm^−1^ and 400 cm^−1^.

#### 2.3.3. X-ray diffractometer (XRD)

The crystal form of the free HK, the HPS, the PS, and PM of HK with PS was detected and analyzed by an X-ray diffractometer (Rigaku Corporation, Tokyo, Japan) with Cu Ka1 radiation at 30 mA and 40 kV. The samples were scanned from 5° to 60° of 2*θ* with a step size of 0.02° at a scan rate of 5°/min.

#### 2.3.4. Differential scanning calorimetry (DSC)

Thermal properties of free HK, the HPS, the PS, and PM of HK with PS were analyzed by using a DSC (TA instruments, New Castle DE, USA). Sample weights varied from 4 to 5 mg and heated from 40 °C to 250 °C at a heating rate of 10 °C/min under a nitrogen purge of 20 mL/min. The DSC instrument was calibrated for temperature with indium. The data were analyzed with Proteus analysis software.

### 2.4. Dissolution study of the HPS

#### 2.4.1. Quantification of HK content

In all the experiments, the quantitative of HK was measured using the high-performance liquid chromatography of WATERS (Waters Corporation, Milford, MA, USA). The separation process was performed on a Diamonsil C_18_ reverse-phase column (250 mm × 4.6 mm, 5 μm, China). The mobile phase consisted of methanol (55%), acetonitrile (20%), and water (25%) with a rate of 1 mL/min. The temperature of the column was maintained at 30 °C. The detection wavelength and the injection volume were set as 294 nm and 10 μL, respectively. All measurements were conducted in triplicates. Then, in the concentration range of 0.5–0.0039 mg/mL, with the concentration of HK as the abscissa and the absorbance as the *y*-coordinate, a linear relation diagram was established, and the regression equation was *y* = 11326308.72*x* − 8679 (*R*^2^ = 0.9999). The retention time of HK was about 10–11 min.

#### 2.4.2. Measurement of equilibrium solubility of the HPS

Apparent solubility was determined by adding an excess of free HK, PM of HK with PS, and the HPS dissolved in buffer solutions with different pH values (1.2(AGJ), 6.8(AIJ), and 5.5), and kept in constant temperature shaker for 48 h at 37 °C. The dissolution mediums contained α-amylase of 1% and glucoamylase of 1%. One milliliter of liquid was were taken out and centrifuged at 10,000 rpm for 10 min, and then filtered through a 0.22 µm membrane filter. The supernatant of the samples was analyzed by the HPLC system after appropriate dilutions in methanol for determining the concentration of HK.

#### 2.4.3. Dissolution study

The *in vitro* release profiles of the HPS, PM of HK with PS, and the free HK were carried out in the above dissolution mediums. The paddle speed and solution temperature were set at 100 rpm and 37.0 ± 0.5 °C, respectively. A total of 30 mg of the free HK and 180 mg of the HPS equating with 30 mg of HK were dispersed in dissolution mediums, and the samples were maintained at 37 ± 0.5 °C with rotation at 100 rpm. At certain time intervals, 1 mL aliquot of the dissolution medium was withdrawn, and the same volume of pre-warmed fresh media was added, respectively. Samples were taken out and centrifuged at 12,000 rpm for 10 min, and then filtered through 0.22 µm membrane filter. The 0.5 mL filtrates were mixed with 0.5 mL methanol for the determination of HK concentrations by the HPLC system. Values were reported as the means for each triplicate sample. The cumulative drug release percentages at each time interval were calculated and plotted against sample collection time to construct the dissolution profile. The cumulative drug release percentages of 24 h were used as the drug release efficiency.

### 2.5. Bioavailability studies

Healthy Sprague–Dawley (SD) rats (200–230 g, *n* = 12) were acclimatized for a week. SD rats were randomly categorized into two different groups having six animals each. Prior to the study, the rats have fasted overnight but with free access to water. The two groups of rats were orally administered by aqueous suspension liquid of free HK and the HPS at the equivalent dose of 50 mg/kg HK, respectively. Blood samples were collected from the retro-orbital plexus at different time intervals (0.08, 0.17, 0.25, 0.33, 0.5, 1, 2, 3, 4, 6, 8, 12, and 24 h.) after dosing and placed into tubes that were pretreated with heparin sodium. These samples were immediately centrifuged, and the plasma samples were stored at −40 °C for further studies.

The animal experiments were implemented according to the Guidelines for Care and Use of Laboratory Animals of Harbin Medical University and approved by the Ethics Committee of the Harbin Medical University. Initially, for 1 week, the animals were acclimatized and maintained at 25 ± 2 °C/50–60% relative humidity in natural light/dark atmosphere before experimentation.

The plasma of 0.2 mL was transferred into a clean 2.0 mL test tube, 0.4 mL of acetonitrile was added, and the mixture was vigorously mixed by vortexing for 30 s to precipitate the protein. After centrifugation at 12,000 rpm for 10 min, the resultant supernatant was transferred to another tube that contained 50 mg of sodium chloride. After vortexing for 30 s, the suspensions were kept for 10 min at 25 °C and centrifuged at 12,000 rpm for 5 min. The supernate was taken out, and the blood sample was again extracted under the same conditions by the methanol. Finally, the extraction obtained was concentrated to dryness under nitrogen, and then dissolved in methanol for analysis by HPLC.

The determination method of HK in plasma samples was as follows: 0.4 mL HK-acetonitrile solutions with different concentrations (15.625, 31.25, 62.5, 125, 250, 500, 1000, and 2000 ng/mL) were mixed with 0.2 mL blank plasma, respectively, and then each sample was treated according to the above operation. Finally, the supernatant of each sample was obtained and determined by HPLC. Using the concentration of HK solution as the abscissa and the absorbency as the *y*-coordinate, the linear chart was constructed, and the regression equation was *y* = 17.157*x* + 41.56 (*R*^2^ = 0.9997). According to the regression equation, the HK concentration in plasma samples at different time points was calculated.

### 2.6. MTT Assay

The inhibition rate of the HPS on HepG2 cell lines was evaluated by conventional methyl thiazole tetrazolium (MTT) cell survival assay. In short, 100 µL cell suspension was placed in 96-well plates, and the plates were pre-cultured in an incubator for 24 h (at 37 °C, 5% CO_2_). Then these cells were incubated with 10 µL of different concentrations of the free HK and the HPS for 48 h (HK content: 100.0, 80.0, 60.0, 40.0, 20.0, 10.0, and 5 μg/mL). The samples containing the HPS and the free HK with different HK concentrations were dissolved with culture medium, and the obtained samples were filtered through a 0.45 μm pore diameter membrane, and then added to the plates. After 48 h, the fresh medium containing MTT was added to each well, and the plates were incubated in an incubator for 4 h. Thereafter, the medium was removed and the formazan crystals were solubilized with DMSO (150 µL). After mild shaking for 10 min, the absorbance value (OD) was detected by the enzyme mark analyzer instrument (detection wavelength of 490 nm and a reference wavelength of 630 nm) and compared with the blank control group.

### 2.7. Statistical data analysis

All data are expressed as the mean ± standard deviation. Statistical significance in the differences of the means was evaluated by using a one-sample *t*-test in the Origin software. For *in vitro* studies, 3–4 replicates were used for each time and three individual experiments were performed and results were averaged. For *in vivo* experiments *n* = 6 was used. A probability value of *p < .05 was considered to be significant.

## 3. Results and discussion

Based on the results of the optimization experiments (Figure S1), 2 h melting time and 5:1 mass ratio of PS to HK were selected as the final optimal conditions regarding the formulation (the HPS) composition and production conditions. The DL and EE of 12.30% and 70.17% were obtained under the optimum conditions, respectively. In addition, the HPS obtained were characterized by SEM, FTIR, XRD, DSC, dissolving capability test, bioavailability test, and antitumor activity evaluation. The analysis results were summarized as follows.

### 3.1. Characterization of the HPS

#### 3.1.1. Micro-morphology analysis

**Figure 2.**
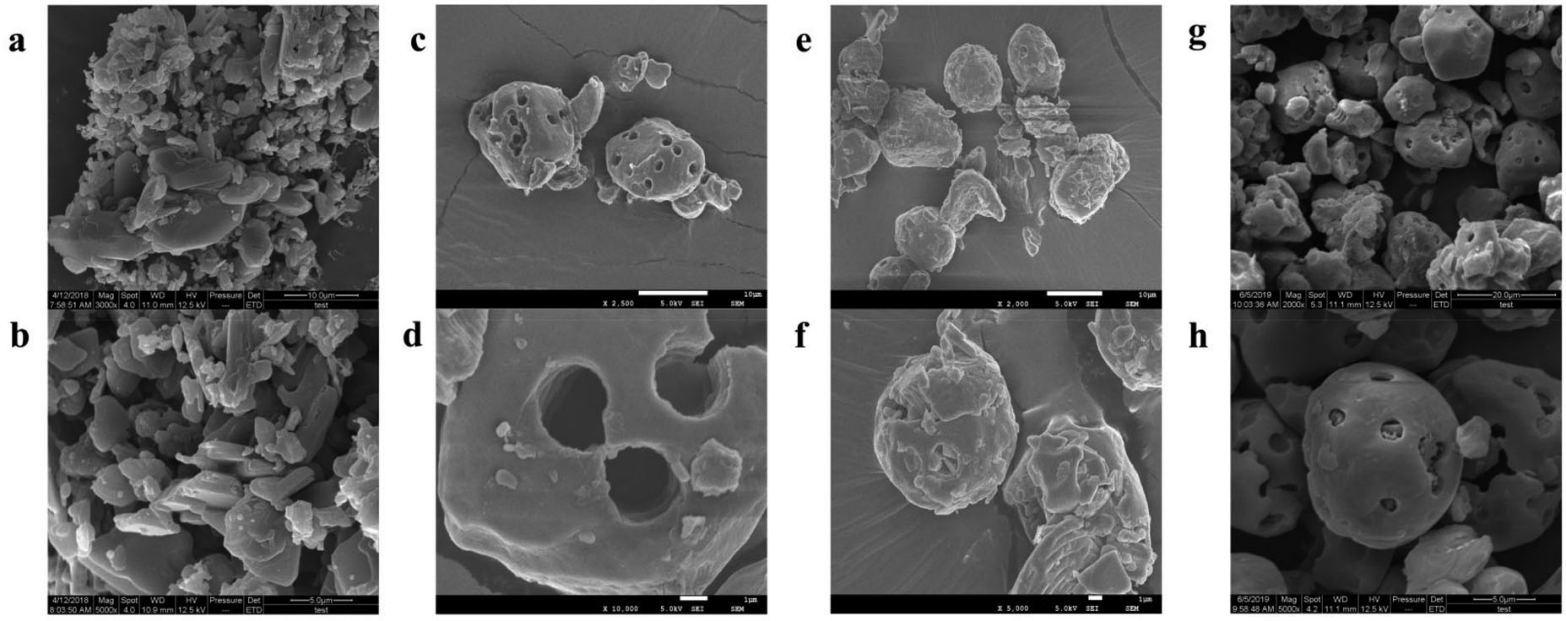
Morphological characterization of the samples by SEM image. Free HK (a, b), the PS (c, d), the PM of HK with PS (e, f) and the HPS (g, h).

SEM images of the free HK, PS, PM of HK with PS, as well as the HPS were illustrated in Figure 2. As shown in Figure 2(a,b), free HK powder was an irregular shape, while the PS (c and d) was nearly spherical, and the circular pores were uniformly distributed on the surface of PS. SEM images (e and f) of the PM showed that the HK were attached to the pore surface and interior of the starch to form a layer, which indicated that proper mixing can increase the chance of contact with HK and PS. However, as can be seen from the SEM images (g and h) of the HPS, the HK was successfully adsorbed inside the pore interior of starch.

#### 3.1.2. FTIR analysis

**Figure 3.**
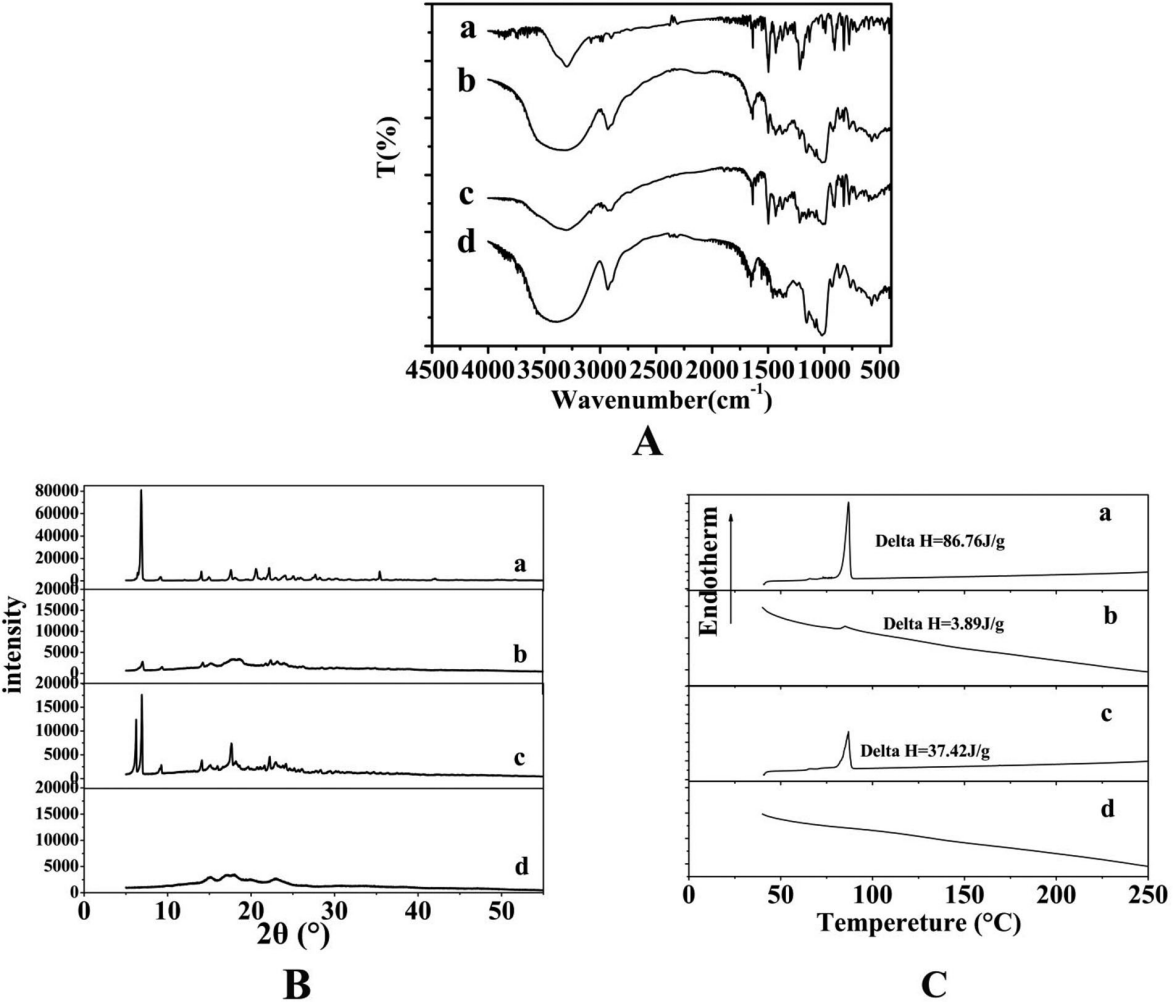
FTIR spectra (Wavenumber: 400–4000 cm^−1^) (A), XRD patterns (2*θ* = 5–60°) (B) and DSC thermograms (Temperature range: 40–250 °C) (C) of free HK (a), the HPS (b), the PM of HK with PS (c), and the PS (d).

The HPS system was further analyzed by FT-IR spectroscopy. The spectra of free HK, PS, PM of HK with PS, and the HPS from 4000 to 400 cm^−1^ were shown in Figure 3(A). The free HK (curve a) exhibited the bands at 3304 cm^−1^, indicating the presence of -OH groups. A band was found at 1638 cm^−1^, which could be assigned to alkene C = C vibration. The intense bands at 1498 cm^−1^ were assigned to C = C aromatic stretching vibration. Moreover, other bands were located at 1217 and 908 cm^−1^ (C–O), 989 and 825 cm^−1^ (C–C). The spectrum of PS was mainly characterized by the intense bands at about 3300–3500 cm^−1^ caused by –OH groups stretching vibration, which was observed in all starches. The band at 2927 cm^−1^ was ascribed to the –CH_2_ stretching vibration. The absorption at 1640–1650.3 cm^−1^ was a typical band that was ascribed to an –OH bending vibration. The absorption bands in the 1300–1500 cm^−1^ region were contributed by the CH deformation vibrations. In the case of PM of HK with PS, the spectrum was basically composed of the overlapping peaks of HK and PS, and the characteristic peaks of the HK almost existed, showing strong absorption, which indicated that most drugs were attached to the starch surface. Compared with the PM of HK with PS, the spectrum of the HPS showed relevant changes in intensities of the typical absorption peaks, demonstrating that most of HK were adsorbed inside the pore interior of the starch. However, a small amount of HK remained coated on the surface of the starch.

#### 3.1.3. XRD analysis

The degree of crystallinity of the HK loaded in the HPS system was evaluated by XRD analysis. Figure 3(B) showed XRD analysis of free HK, the PS, PM of HK with PS, and the HPS. As Figure 3(B) shows, the free HK exhibited several sharp diffraction peaks at the range of 5°–35°, suggesting that the HK exhibited a crystalline structure. The XRD pattern of PS showed three broad peaks at the range of 14°–16°, 16°–19°, and 21°–25°, confirming that the PS was amorphous (Jiang et al., 2017). Moreover, the characteristic peaks of PM of HK and PS were similar to the superposition of characteristic peaks of HK and PS, and the characteristic peaks of the HK almost all existed. However, the HPS sample (1/5 HK/PS) had a markedly lower intensity than the physical mixture with the same ratio and the characteristic peaks of the HK almost disappeared, illustrating that crystallization of the HK was significantly reduced by adsorption of the PS and the HK was almost amorphous in the HPS, which was beneficial to the increase of solubility and dissolution rate of the HK.

#### 3.1.4. DSC analysis

Figure 3(C) showed the thermostability of the HPS system through DSC analysis under N_2_ atmosphere. From this figure, the free HK showed a clear and sharp endothermic peak at 86.7 °C, which represented the melting point of HK, indicating that HK existed in the form of crystals in nature. The PS had no obvious endothermic peak, which indicated that the PS was amorphous thus do not undergo any thermal transition and the PM of HK with PS showed endothermic transition similar to free HK with lesser intensity because of the possible dilution with PS. However, the HPS sample (1/5 HK/PS) has a weaker melting peak than the PM with the same ratio, which might be due to most of HK being adsorbed inside the pore interior of the PS. In addition, the endothermic amount of the HPS was about 3.89 J/g, which was significantly lower than compared with that of the free HK (86.76 J/g) and the PM of HK with PS (37.42 J/g). The endothermic peak of the HPS was attributed to HK. Thus, combining the results of XRD and DSC, it could be further confirmed that the HK in the HPS was mostly amorphous.

### 3.2. Solubility and dissolution studies of the HPS

#### 3.2.1. Solubility study

**Figure 4.**
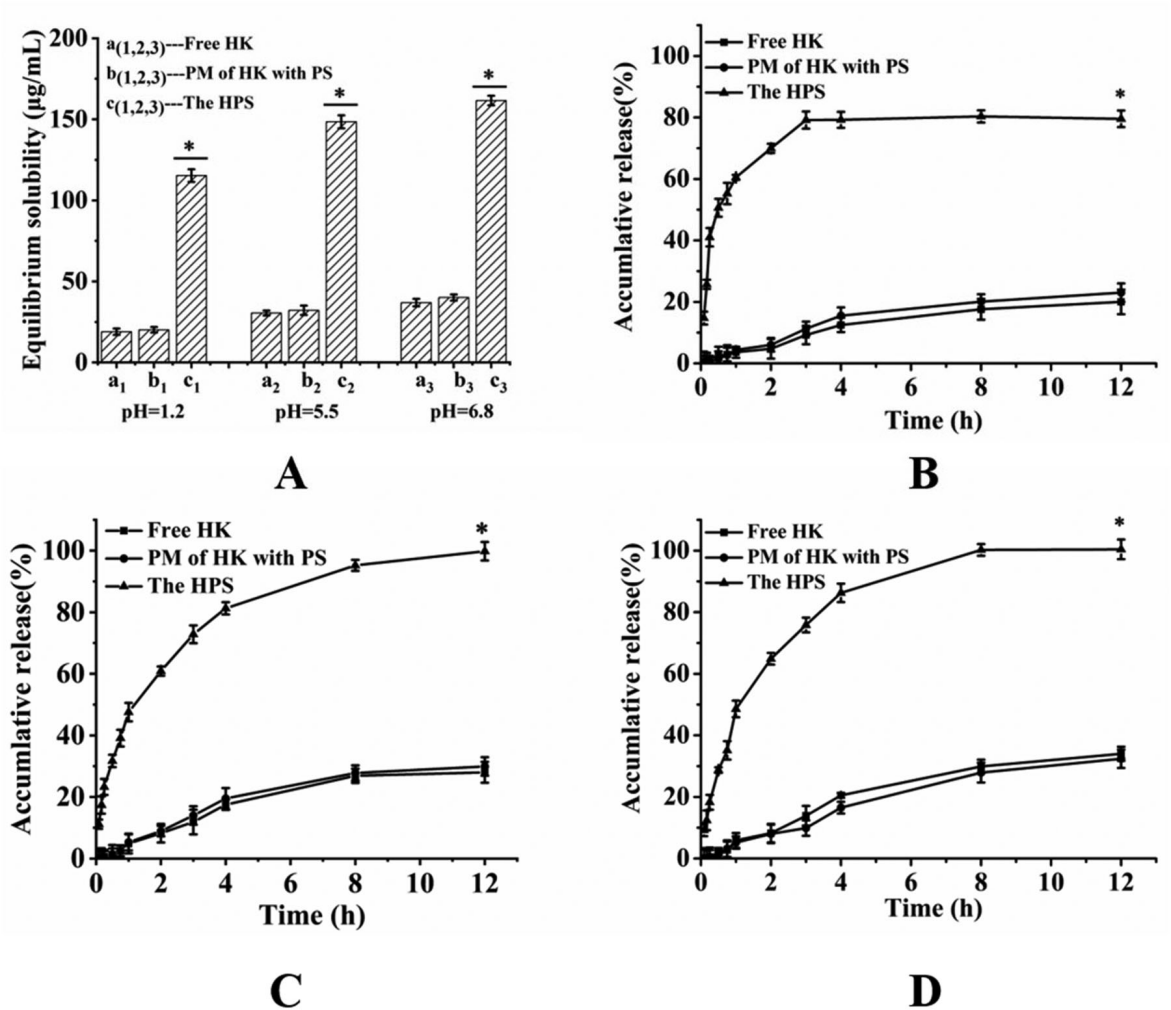
Equilibrium solubility (A) for free HK (a_(1,2,3)_), the PM of HK with PS (b_(1,2,3)_), and the HPS (c_(1,2,3)_)in buffer solutions with different pH value (1.2, 5.5, and 6.8); Data are presented as mean ± standard deviation (*n* = 3)(*p < .05); The release profiles of free HK, the PM of HK with PS and the HPS in buffer solutions with different pH value (1.2 (B), 5.5 (C), and 6.8 (D)) by different time intervals. Data are presented as mean ± standard deviation (*n* = 3) (*p < .05).

The solubility of HK in buffer solutions with different pH values (1.2, 5.5, and 6.8) was investigated using free HK, PM of HK with PS, and the HPS (Figure 4(A)). The solubility of free HK was about 18.98 ± 2.32 (pH = 1.2), 30.5 ± 1.63 (pH = 5.5), and 36.88 ± 3.24 (pH = 6.8) μg/mL, respectively, while that of the PM of HK with PS was 20.12 ± 3.21 (pH = 1.2), 32.2 ± 2.01 (pH = 5.5), and 40.1 ± 1.92 (pH = 6.8) μg/mL, respectively, which was not different from that of free HK. The difference between the two groups was not statistically significant (*p* > .05). This showed that the PS exerted little or no solubility enhancing effect. Nevertheless, the solubility of the HPS by the HPLC detection was about 115.27 ± 2.92 (pH = 1.2), 148.5 ± 1.89 (pH = 5.5), and 161.58 ± 3.42 (pH = 6.8) μg/mL, was obviously higher than the others, indicating that the crystallinity of the HK decreased after loading into the PS, so that the HK had a higher solubility. The difference between the HPS group and the free HK group was statistically significant (*p* < .05). So, the HPS could have better dissolution and bioavailability.

#### 3.2.2. In vitro dissolution study

To evaluate the dissolution characteristics of HK from the HPS for oral administration, the buffer solutions with different pH values (1.2, 5.5, and 6.8) were used as the dissolution medium. From Figure 4(B, C and D), it can be seen that the free HK displayed a poor dissolution profile and less than 35% of HK was finally dissolved in each buffer solution, while about 32.4% (pH = 1.2), 29.9% (pH = 5.5) and 23.02% (pH = 6.8) of HK dissolved in buffer solutions from the PM within 12 h, respectively. It could be seen from the results that there was no significant difference in the dissolution rate between the free HK and the PM, which might be due to the fact that the PS exerted little or no solubility enhancing effect. The difference between the two groups was not statistically significant (*p* > .05). However, the HPS showed a more rapid dissolution rate with a much higher cumulative amount of dissolved HK in each buffer solution within 12 h, the cumulative release rate of the HPS was about 55.27% (pH = 1.2), 39.13% (pH = 5.5) and 35.13% (pH = 6.8) in each buffer solution within 0.75 h, respectively. Eight hours later, the dissolution rate of the MPS was basically balanced and the cumulative release rate of the HPS were 79.55% (pH = 1.2), 99.8% (pH = 5.5), and 100% (pH = 6.8) in each buffer solution, respectively at 12 h. The difference between the HPS group and the free HK group was statistically significant (*p* < .05). The results indicated that the amorphous HK released from HPS was steady and rapid, and was significantly better than that of the crystalline-free HK and the PM. Thus, the HPS might have better oral bioavailability than the free HK.

### 3.3. Pharmacokinetic behavior of PSN in rats

**Figure 5.**
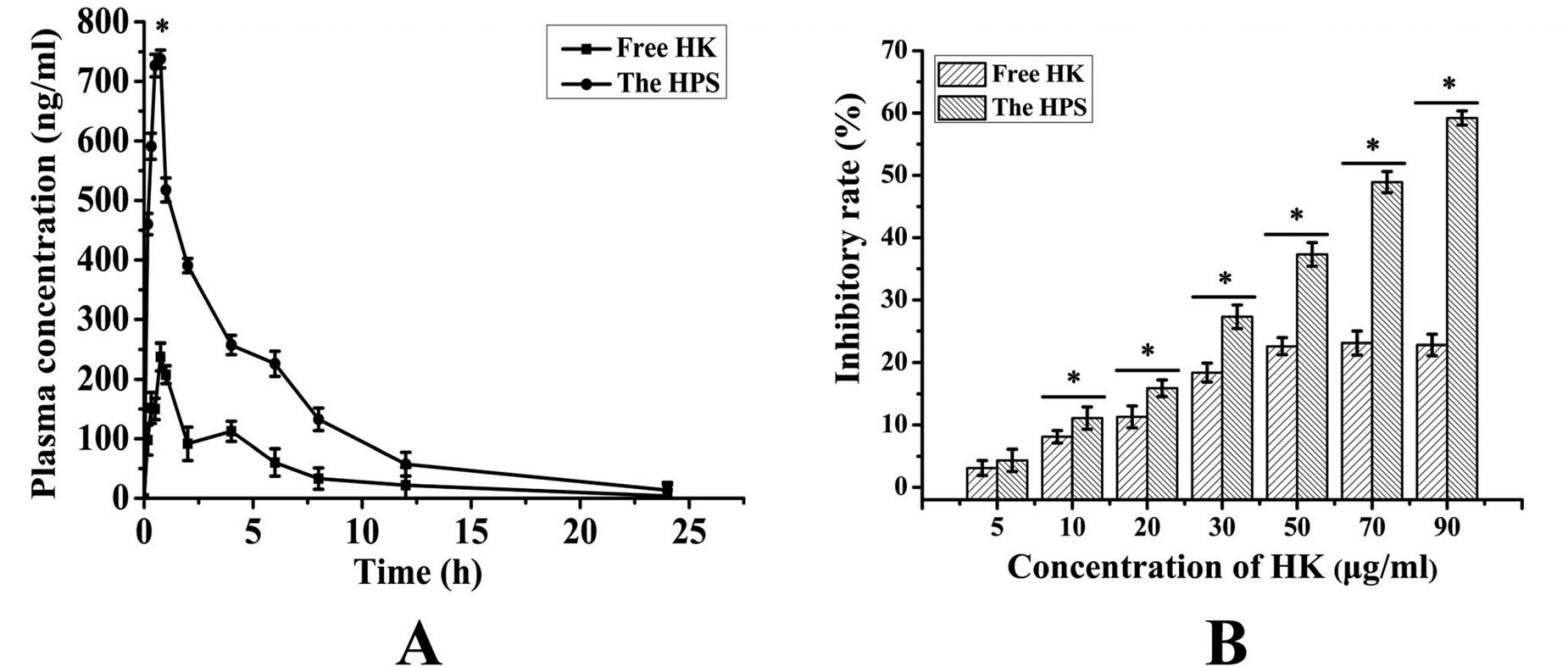
The bioavailability result (A) of the free HK and the HPS in rats. Data are presented as the mean ± standard deviation (*n* = 6). *p < .05 vs. free HK; The viability diagram (B) of different amount of the free HK and the HPS (HK content: 100.0, 80.0, 60.0, 40.0, 20.0, 10.0, and 5 μg/mL) on HepG2 cells after 48 h of treatment. Data are presented as mean ± standard deviation (*n* = 3). *p < .05 (vs. free HK).

After oral administration, the mean plasma concentration-time profiles for the HPS and free HK were presented in Figure 5(A). The results demonstrated that both the HPS and free HK concentration–time curves could be fitted to the two-compartment model. The relevant pharmacokinetic parameters for the compartmental analysis were listed in Table 1. As shown in Figure 5(A) and Table 1, the plasma concentration of the HK was remarkably improved by the HPS delivery system at each predetermined time point. The difference between the HPS group and the free HK group was statistically significant (*p* < .05). The plasma concentration of the HK reached the maximum concentration (*C*_max_) of 737.76 ± 4.34 ng/mL after oral administration of HPS, which was obviously increased 3.11-fold over that of the free HK (237.35 ± 3.98 ng/mL). Moreover, the AUC (0−*t*) value of HPS (3406.196 ± 8.33 ng/mL h) was significantly increased 3.51-fold than that of free HK (971.608 ± 11.39 ng/mL h). Therefore, these results showed that the oral bioavailability of the HPS was improved significantly compared with the free HK. The significant improvement of oral bioavailability of the HPS might be due to the decrease of the crystallinity of the HK, which could be seen from the XRD diagram.

**Table 1.** Pharmacokinetic parameters for the HK in rats after oral administration of free HK and the HPS.

Pharmacokinetic parameters	Free HK	The HPS
*C*_max_ (ng/mL)	237.35 ± 3.98	737.76 ± 4.34
*T*_max_ (h)	0.75 ± 0.13	0.75 ± 0.11
*t*_1/2_ (h)	0.357 ± 0.12	2.834 ± 0.23
MRT (0–*t*) (h)	5.334 ± 1.33	5.191 ± 1.23
MRT (0–∞) (h)	5.923 ± 1.98	5.889 ± 1.63
AUC (0–*t*) (ng/mL h)	971.608 ± 11.39	3406.196 ± 8.33
AUC (0–∞) (ng/mL h)	1003.996 ± 13.22	3538.057 ± 9.46

### 3.4. MTT Study

HepG2 cells were treated with different concentrations of the HPS and the free HK, and cell viability was assayed at 48 h using the MTT assay. As shown in Figure 5(B), both the HPS and the free HK have certain inhibitory effects on HepG2 cell growth, and with the increase of drug concentration, the ability to inhibit HepG2 cell growth was gradually increased. When the concentration of the free HK exceeded 60 μg/mL, the inhibition rate was no more significantly increased, and the maximum inhibitory rate was 22.6 ± 2.12%. Nevertheless, the HPS showed more effective inhibition than free HK and the maximum inhibitory rates of the HPS reached 59.2 ± 3.15%, and the difference between the groups was statistically significant (*p* < .05), which could be explained by the increased cellular uptake of the HPS formulation. The above results suggested that the inhibitory effect of the HPS was better than that of the free HK.

## 4. Conclusion

In this paper, we first attempt to prepare the HK-loaded PS system by the melting method without using organic solvents. The single-factor experiments were used to optimize the procedure for the preparation of the HPS, and the preparation conditions obtained were: The best mass ratio of PS to HK was 5, and the best melting time was 2 h. Then, the physicochemical properties of the HPS were investigated, and the results showed that the HK was successfully adsorbed inside the pore interior of the PS and the HK in the HPS was mostly amorphous. The solubility of the HPS was 6.07 (pH = 1.2), 4.87 (pH = 5.5), and 4.38 (pH = 6.8) times as much as that of free HK in the corresponding environments respectively. Moreover, the release rate of the HPS was also obviously better than that of free HK in each buffer solution. *In vivo* pharmacokinetics showed that the *C*_max_ and the AUC (0–*t*) values of the HPS were approximately 3.11 and 3.51-fold greater than the free HK. MTT study showed that the HPS generated a higher inhibition to HepG2 cells than free HK. It can be therefore concluded that the HPS prepared could dramatically enhance the absorption of HK *in vivo*, and possess potential clinical application value as a new oral drug formulation. Furthermore, this work presented for the first time an HPS system produced by the melting method with potential for oral drug release, and this method has obvious advantages in cost and manufacturing feasibility and also provides a new idea for lipophilic drugs with a low melting point.

## Supplementary Material

Supplemental Material

## Data Availability

Data sharing not applicable to this article as no datasets were generated or analyzed during the current study.
