# Supplementary material for: Construction, characterization, and bioavailability evaluation of honokiol-loaded porous starch by melting method without any solvent
Source: Drug Deliv. 2021 Dec 6;28(1):2574–81. doi: 10.1080/10717544.2021.2009938 (PMC8654402; doi:10.1080/10717544.2021.2009938)
Supplement: Supplemental Material [file IDRD_A_2009938_SM7530.doc]

Supplementary Material

**Title:** Construction, characterization and bioavailability evaluation of honokiol-loaded porous starch by melting method without any solvent

Weiwei Wu *, Haiyang Xu

School of Basic Medical Sciences, Shandong First Medical University & Shandong Academy of Medical Sciences, Taian 271000, Shandong, China

* Corresponding author: Weiwei Wu

E-mail address: wuweiwei0522@163.com (Weiwei Wu).

***1. Optimization of the preparation process***

In this experiment, the HPS samples were prepared by melting method. Because there was no drug loss in the preparation process and all drugs were in the systems, the actual drug loading (DL) could not be calculated. Therefore, the DL of the HK-loaded PS system was calculated according to the initial quality of HK and PS, that was DL= (*W_HK_* /*W_HPS_*)×100%( *W_HK_* was the initial amount of HK (mg) and *W_HPS_* was the total weight of HPS (mg)). In addition, in the preparation processes, the mass ratio of PS to HK and the melting time can affect the solubility of the HPS samples. Therefore, the single-factor experiment was operated to investigate the effects of the two factors on the solubility of the HPS, so as to obtain the optimal operating conditions. Through preliminary experiments, the value of each factor was preliminarily confirmed, as follows: The mass ratio of PS to HK was studied at 2.5-15. The melting time was studied at 0.5-4 h. The melting temperature was fixed to 90°C. Finally, the optimum condition was obtained based on the solubility of the HK. The experiment was conducted in triplicate. The sample treatment was as follows: the HPS samples were prepared by melting method under different conditions. Each HPS sample (the content of HK was excessive and consistent) was weighed and placed in small beakers with 4 mL of deionized water, and then the beakers were sealed and placed in a water bath at 37 ± 1.0 °C at a rotation speed of 100 rpm. After 6 h, the samples were centrifuged at 12 000 rpm for 10 min, and then filtered through 0.22 um membrane filter to remove the possible residual drugs. The supernatant obtained was used to HPLC to determine HK concentration.

In this experiment, we mainly used the melting method to prepare the HPS samples. Because there was no obvious loss of drug in the preparation process, and almost all drugs were in the system, the preparation conditions had no significant effect on the drug loading. Therefore, we mainly investigated the influence of preparation conditions on the solubility of the HPS samples by the single-factor experiments, including the mass ratio of PS to HK and the melting time, so as to obtain the best operation conditions. The melting temperature was fixed at 90°C, which was slightly higher than the melting point of the HK. The experimental results were shown in Figure S1.

As can be seen from the Fig. S1(a), with the mass ratio of PS to HK increasing from 2.5 to 5, the solubility of the HK increased from 42.44 to 61.54 μg/mL. However, when the mass ratio of PS to HK was higher than 5, the solubility of the HK scarcely changed. The results can indicate that the quality of PS increased with the mass ratio of PS to HK. The more PS content, the more pores, and the more HK can get into the pore. After the HK entered the pores, its particle size decreased, the specific surface area increased, and the crystallinity decreased, resulting in the increase of its solubility. However, when the PS content further increased, the solubility of the HK changed little, which indicated that when the PS content ensured that the drug fully entered into its pores, the amount of starch increased further, and the effect on its solubility was not significant. Therefore, the mass ratio of PS to HK (5) was selected as the optimum condition.

As can be seen from the Fig. S1(b), with the melting time increasing from 0.5h to 2h, the solubility of the HK increased. When the melting time was 2h, the solubility of the HK was the highest at about 62.15 μg/mL. But when the melting time was more than 2 h, the solubility of the HK almost unchanged. This might be because when the melting time was less than 2 h, the HK powder might not melt completely, which was not conducive to the drug entering the pores of the PS. Therefore, the melting time (2 h) was selected as the optimum condition.

Therefore, 2 h melting time and 5:1 mass ratio of PS to HK were selected as the final optimal conditions regarding the formulation composition and production conditions. The DL and EE of 12.30% and 70.17% were obtained under the optimum conditions, respectively.

***
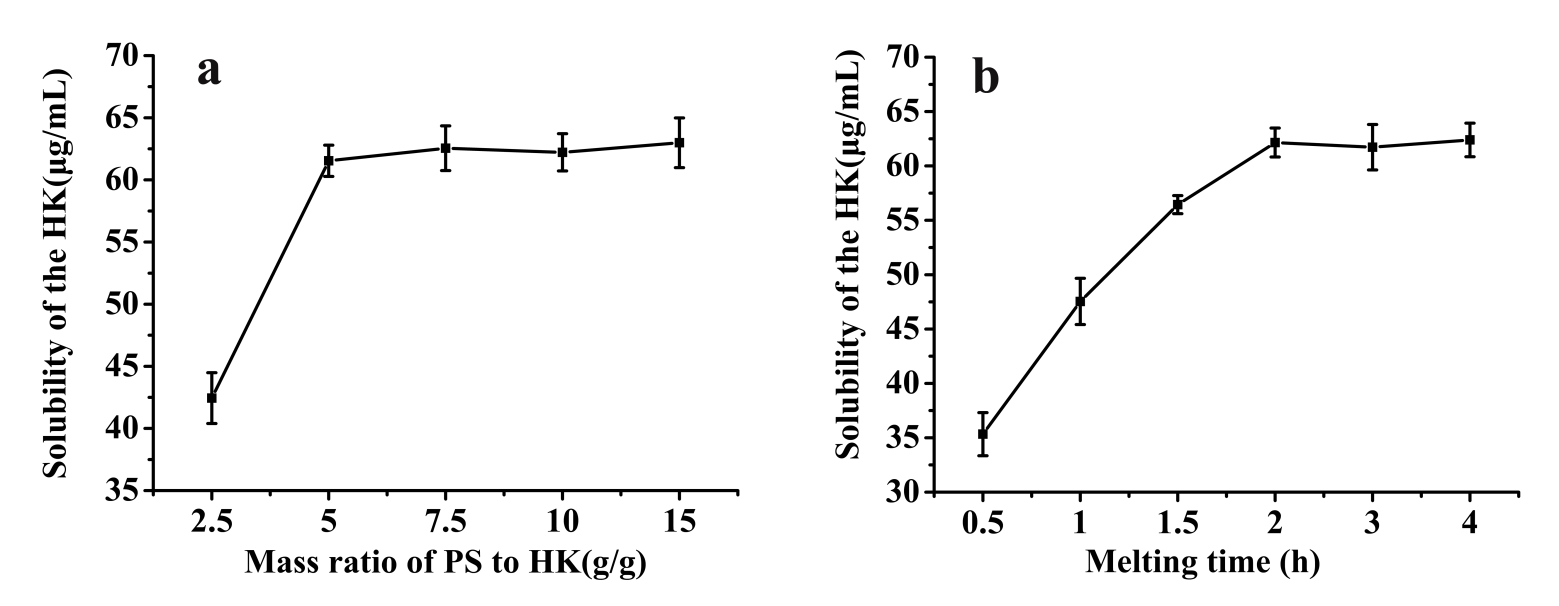
***

Fig.S1. Influence of (a) mass ratio of PS to HK and (b) meiting time on the solubility of the HPS.
